# Harnessing Folate-Functionalized Nasal Delivery of Dox–Erlo-Loaded Biopolymeric Nanoparticles in Cancer Treatment: Development, Optimization, Characterization, and Biodistribution Analysis

**DOI:** 10.3390/ph16020207

**Published:** 2023-01-30

**Authors:** Ms Farheen, Md Habban Akhter, Havagiray Chitme, Md Sayeed Akhter, Fauzia Tabassum, Mariusz Jaremko, Abdul-Hamid Emwas

**Affiliations:** 1 School of Pharmaceutical and Population Health Informatics (SoPPHI), DIT University, Dehradun 248009, India; 2 Department of Clinical Pharmacy, College of Pharmacy, King Khalid University, Abha 62529, Saudi Arabia; 3 Department of Pharmacology, College of Dentistry and Pharmacy, Buraydah Private College, Buraydah 51418, Saudi Arabia; 4 Smart-Health Initiative (SHI) and Red Sea Research Center (RSRC), Division of Biological and Environmental Sciences and Engineering (BESE), King Abdullah University of Science and Technology (KAUST), Thuwal 23955, Saudi Arabia; 5 Core Labs, King Abdullah University of Science and Technology (KAUST), Thuwal 23955, Saudi Arabia

**Keywords:** brain targeting, nanoparticles, folate receptor, glioma cancer, doxorubicin, erlotinib, blood–brain barrier

## Abstract

The aim of the present study is to develop Doxorubicin–Erlotinib nanoparticles (Dox–Erlo NPs) and folate-armored Dox–Erlo-NP conjugates for targeting glioma cancer. Glioma is one of the most common progressive cancerous growths originating from brain glial cells. However, the blood–brain barrier (BBB) is only semi-permeable and is highly selective as to which compounds are let through; designing compounds that overcome this constraint is therefore a major challenge in the development of pharmaceutical agents. We demonstrate that the NP conjugates studied in this paper may ameliorate the BBB penetration and enrich the drug concentration in the target bypassing the BBB. NPs were prepared using a biopolymer with a double-emulsion solvent evaporation technique and functionalized with folic acid for site-specific targeting. Dox–Erlo NPs and Dox–Erlo-NP conjugates were extensively characterized in vitro for various parameters. Dox–Erlo NPs and Dox–Erlo-NP conjugates incurred a z-average of 95.35 ± 10.25 nm and 110.12 ± 9.2 nm, respectively. The zeta potentials of the Dox–Erlo NPs and Dox–Erlo-NP conjugates were observed at −18.1 mV and −25.1 mV, respectively. A TEM image has shown that the NPs were well-dispersed, uniform, de-aggregated, and consistent. A hemolytic assay confirmed hemocompatibility with the developed formulation and that it can be safely administered. Dox–Erlo-NP conjugates significantly reduced the number of viable cells to 24.66 ± 2.08% and 32.33 ± 2.51% in U87 and C6 cells, respectively, and IC50 values of 3.064 µM and 3.350 µM in U87 and C6 cells were reported after 24 h, respectively. A biodistribution study revealed that a significant concentration of Dox and Erlo were estimated in the brain relative to drug suspension. Dox–Erlo-NP conjugates were also stable for three months. The findings suggest that the developed Dox–Erlo-NP conjugates may be a promising agent for administration in glioma therapy.

## 1. Introduction

Glioma results from the growth of malignant tissue in the brain or spinal cord and is extremely difficult to treat. Any drug targeting glioma must overcome the blood–brain barrier and effectively target any of a variety of cells proliferating at different rates while also providing a satisfactory safety profile. Gliomas destroy surrounding tissue and are associated with a devastating loss of functionality and a poor prognosis, with the mean survival rate from the time of diagnosis being <2 years [1]. The tumors show a limited response to conventional chemotherapy, and the development of therapies to specifically target the malignant cerebral or spinal tissue is extremely challenging due to the nature of the blood bran barrier (BBB) and the wide variety of malignant cells, different locations of tumors, and high rates of cell proliferation. Current therapeutic approaches are based on neurosurgical procedures, advanced radiotherapy, and a variety of emerging chemotherapies. Among novel drug therapies, nano-particle compounds are the most promising as these formulations penetrate the blood–brain barrier (BBB) and the blood–brain tumor barrier (BBTB) more effectively than conventional drugs. They allow for more selective tumor targeting as well as a reduction in the size and frequency of dosage, thus improving the options for the development of tailored formulations and less invasive therapies for this patient population. When successful, targeted drug therapies that are tailored to the specific malignancies of individual patients will provide hope to this patient population as a whole, promising to meaningfully extend their individual lifetimes following a glioma diagnosis [1].

Despite advances in nanotechnology and the development of multimodal therapies, disease prognosis remains a main challenge for therapeutic, drug-based interventions. Gliomas in the brain represent 57% of all gliomas, while 48% are malignancies associated with the central nervous system [2].A range of tumors may develop within each category, each requiring tailored intervention. The current standard of therapy for gliomas includes chemotherapy and radiotherapy with temozolomide, a combination of radiation and chemotherapy followed by surgical resection [3]. The surgery is carried out to excise the tumor; however, successful excision is no guarantee against re-growth or metastasis. The invasive nature of surgical procedures is also associated with risk to surrounding tissue, and the development of efficient, effective, and safe drug-based therapies tailored to target individual tumors is therefore highly desirable and may lead to a real improvement in survival rates and quality of life for this patient population [4].

Conventional chemotherapy is inefficient in treating gliomas because of the two barriers in the brain: the blood–brain barrier (BBB), and the blood–brain tumor barrier (BBTB). These barriers limit the transportation of dissolved, active therapeutic agents to the brain while also inhibiting drug excretion, i.e., the removal of metabolites and drug residues via the blood stream, thus limiting the effects on the tumor while also increasing the risk of damage to other, healthy tissue. Chemotherapeutic agents are toxic formulations known to cause multiple adverse responses due to their lack of tissue specificity, the high doses required to successfully target malignant tissue, and the required frequency of dosage. In addition, the limited excretion of metabolites and drug residues from the treated tissues leads to drug deposition and the accumulation of damaged tissues in the normal and neighboring cells/tissues, adding to the toxic burden and exacerbating the harmful effects of already toxic drugs [5,6].

In accordance with estimates for 2021, 83,570 people in the U.S.A. alone were expected to be diagnosed with a brain tumor. Of this number, 24,530 were expected to be malignant tumors and 59,040 were expected to be non-malignant tumors, with brain tumors being established as the likely cause of death in 18,600 of these cases [7]. More alarmingly, the most recent evidence suggests that there was a massive growth in the global occurrence of glioblastoma between 1995 and 2015, with more than double the rate of 2.4 to 5.0 per 100,000 individuals in the U.K. Glioblastoma occurrence is predicted to also increase dramatically in the U.S.A. over the next 30 years [8].

Erlotinib [N-(3-ethynylphenyl)-6,7-bis(2-methoxyethoxy)-4-quinazolinamine] is a quinazoline compound with antineoplastic activity that functions as an epidermal growth factor receptor (EGFR) antagonist and protease inhibitor. The main action mechanism of this drug is the inhibition of the phosphorylation of tyrosine kinase associated with tumor growth. However, this compound has a limited ability to overcome the constraints of the BBB and BBTB; it may therefore not be a suitable alternative for glioma patients [9].

Doxorubicin (Dox) is one of the most commonly recommended agents for the treatment of benign and malignant tumors, including solid and liquid tumors. Its action mechanism involves the inhibition of the topoisomerase II inhibitor, leading to a transient arrest of the cell cycle in the G2 to M phases. However, as is the case with quinazoline, this drug has limited BBB and BBTB penetrability [10].

In recent decades, nano-scale technology has emerged as a useful tool in a range of sciences and industrial applications, including pharmacology and the production of pharmaceuticals [11]. The size of nanoparticles (NPs) generally varies in the range of 1–100 nm, thus meeting one criterion for successful drug penetration of the BBB and the BBTB. As drug carriers, nanoparticles protect therapeutic agents from degradation in the biological fluid, provide bio-stability, prevent early release, and enable the transport of drug compounds to the intended tissues/cells [12]. The surface area and mass ratio of NPs are higher than macro-scale particles, resulting in the unique features of very small size and high drug-loading/encapsulation capacities, making nanoparticles important candidates as carriers of both diagnostic and therapeutic agents [13,14]. NP technologies applied to biomedical sciences enable the tailoring of drugs to specific tissues in a controlled manner, thus opening up the development of drug tailoring for individual patients and novel diagnostic tools [15]. So far, NPs have demonstrated advantages in the targeting of glioma tumors by enabling BBB and BBTB penetration for site-specific delivery, enabling precise drug-to-tissue tailoring, minimizing off-target effects, and reducing drug dose and frequency, and reducing the duration of drug administration, which also helps to improve patient compliance [16,17].

The BBB was first identified by Ehrlich in 1885 through a dye test. It comprises endothelial cells such as astrocytes, pericytes, and neuronal cells. Endothelial cells primarily restrict the passive transport of substances from blood to the brain. The permeability of brain blood vessels can be increased only when the BBB is ruined; however, some blood vessels nurturing a tumor form the BBTB. The BBTB causes less hindrance to the transport of substances in a brain tumor. The blood vessels bear over-expressed receptors, which could facilitate the ligand-gated active targeting of substances in the tumor microenvironment. To overcome the BBB and achieve a successful therapeutic delivery in the brain, ligand-targeted NPs have an overwhelming response in the diagnosis and treatment of glioma [18]. Luque-Michel et al. injected mice with polymeric NPs loaded with both superparamagnetic iron oxide NPs and Dox and, using an MRI, observed the high accumulation of nanocarriers in a glioma tumor region, leading to the successful suppression of tumor growth [19].

It is well established that the folate receptors (FR) are over-expressed in a range of solid tumor cells, viz., non-small cell lung cancers, colorectal, pancreatic, ovarian, breast, kidney, gastric, and prostate cancers, including glial tumors of the brain and central nervous system [20]. Folate receptors are glycoprotein-based receptors with a molecular weight in the range of 38–45 kDa, and folic conjugate has been proven to have higher rates of uptake than conventional therapies through folate-receptor-mediated endocytosis in tumor cells [21]. A high level of expression of folate receptors has been observed in solid tumors of the body. These sites may therefore represent an ideal target domain for nanocarriers [22]. In the development of drugs, folic acids (FA) are biotechnological ligands as they retain a high affinity for folate receptors and enable the targeted delivery of drugs to a tumor. Like antibodies, FAs are superior targeting ligands due to their relatively smaller size and lack of immunogenicity. They are readily available and have relatively simple conjugation properties. Folic acids have been used for a while as targeting ligands for nanoparticle uptake in cancer cell lines and are extensively explored as a targeting ligand for cell lines and tumors that over-express folate [23].

A biopolymer is a macromolecule composed of repeating structural units of monomers with covalent bonds that form a chain-like structure. Recently, biopolymers have gained wider attention with a view to develop pharmaceutical nanocomposites meeting the essential requirements of having antimicrobial properties: having stability and flexibility and being biocompatible, biodegradable, and bioresorbable [24]. Biopolymers have the major advantage of being easily broken down in the biological system by naturally occurring microorganisms and enzymes. Additionally, as their by-products are organic and without detrimental impact on the biological system, they are highly promising carriers for therapeutic drugs. As a result, a significant body of work has emerged on polysaccharide-based biopolymers for biomedical applications, replacing synthetic nanomaterials to hopefully improve efficacy and safety profiles and reduce the harmful side effects of anti-cancer drugs.

In the present study, biopolymers obtained from a natural source—the bark of *Cinnamomum zeylanicum*—were used to achieve drug encapsulation and the delivery of the active compound to the targeted site, facilitating its entry into the brain tumor [25]. This is the first time we have prepared a combined formulation of Erlo and Dox in functional biopolymer. We expect this formulation to have the dual advantage of diminishing resistance development in cancer cells and eliciting ameliorating, anti-cancer effects via the inhibition of epidermal growth factor receptor (EGFR) and damage to DNA. The functionalized nanoparticles were delivered via a naturally obtained *Cinnamomum zeylanicum* biopolymer, while the formulation was evaluated in vitro for physicochemical characteristics such as particle size, surface charge, % drug release. The biocompatibility of NPs was assessed using a hemolysis assay. We also examined the formula’s biodistribution and cell viability against glioma cell lines and performed stability studies in vitro.

## 2. Results

### 2.1. Formulation Optimization

The experimental design applied a novel tool to achieve statistical optimization and enable the minimization of method-induced variability while yielding a high-quality product with uniform and homogeneous particle size distribution, as well as methodical stability for other parameters under study. As in many other experimental designs, the Box–Behnken design was used to optimize and investigate the principal effects, interactions, and quadratic effects of the independent variables on responses, viz., particle size, PDI, and %drug release. This design is effective for exploring quadratic response surfaces and constructing second-order polynomial models [26]. The Dox–Erlo NPs were optimized using the Box–Behnken design and a preliminary formulation was developed based on the trial-and-error method to identify the desirable components and select the appropriate concentration for the independent variables. According to this examination, a surfactant concentration lower than 1.00% *w*/*v* yielded larger NP sizes owing to a minimum size reduction attributed to poor emulsification, resulting in low drug encapsulation and impaired drug release. Furthermore, NPs in the >3.00% *w*/*v* size range demonstrated a diminished overlay and an extremely poor drug profile. To obtain the required particle sizes for the formulation, sonication below 3.00 min of sonication time is required. Size reductions of the nanoparticles were observed to be low. Above 12.00 min small particles were in line to accumulate, leading to instability issues, possibly due to an excess reduction of particle size. Thus, the selection of low and high levels of excipient concentrations was based solely on primary investigations. In this context, the surfactant levels were low (−1), medium (0), and high, (+1); at 0.50, 1.50, and 2.50% (*w*/*v*), respectively. Polymer concentrations were 1.00, 2.00, and 3.00% (*w*/*v*); and the sonication time levels were low, (−1), medium (0), and high (+1); corresponding to 3.00, 7.50, and 12.00 min, respectively, as is depicted in Table 1. The obtained independent variable data in the Box–Behnken design and their corresponding responses according to experimental runs are shown in Table 2.

**Figure 1:**
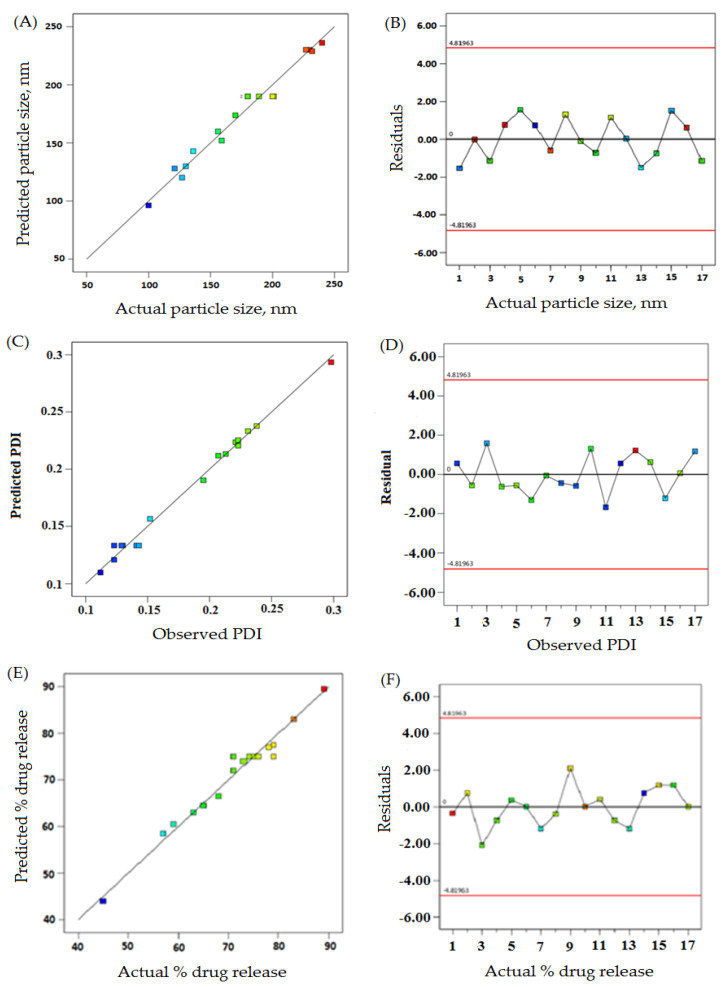
The actual vs. predicted values represented as linear correlation plots (**A**,**C**,**E**), and associated residual plots (**B**,**D**,**F**) providing responses according to particle size, PDI, and %drug release.

**Figure 2:**
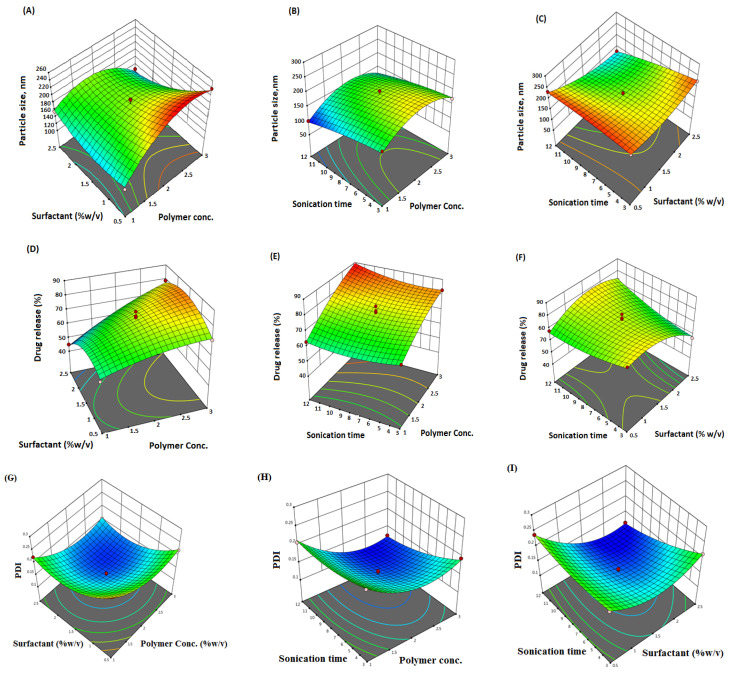
Three-dimensional (3D) surface response plot (**A**–**F**) indicating comparative effects of polymer, surfactant, and sonication time on responses, particle size (**A**–**C**), % drug release (**D**–**F**), and PDI (**G**–**I**).

The linear correlation plots (A, C, E) and their residual plots (B, D, F) between actual vs. predicted values of particle size, PDI, and % drug release, are indicated in Figure 1. Fitting data to the various models—viz., cubic, 2FI, linear, and quadratic—in the Box–Behnken design indicated the quadratic model for each response. The best-fitted model for each response was selected using ANOVA by regression analysis the calculation of F values. The response surface morphology of the BBD expresses the individual, combined, and quadratic impacts on the dependent variables, viz., particle size (nm), PDI, and % drug release (Figure 2). The outcome of the regression analysis for particle size (R1), % drug release (R2), and the PDI (R3) of formulation are provided in Table 3. The analysis of variance of the calculated models for responses are shown in Table 4.

### 2.2. Response 1: Effect on Particle Size

The effects on particle size of various excipients used in the formulation are explained by the quadratic equation (Table 2).In the above equation of particle size (Table 2), the terms A, B, C, AB, AC, BC, A^2^, B^2^, and C^2^ are significant. The model’s F-value, 32.68, suggested a significant model. The statistical *p*-values < 0.05 indicate significant model terms, while *p* > 0.05 indicates insignificant model terms. The lack-of-fit F-value of 0.85 expressed remained insignificant for this quadratic model.

The size of particles in the formulations were reported from 100 to 240 nm in formulations number 6 to 3 (Table 2). The surfactant concentration revealed positive and negative effects on particle size. For example, formulations number 2 and 4 exhibited particle sizes of 230 nm and 240 nm, respectively, at a 0.5% concentration of surfactant. On the other hand, a particle size of 227 nm at a 2.5% concentration of surfactant was found for formulation number 7. Formulation number 6 and formulation number 1 demonstrated a particle size of 100 nm and 121 nm, respectively, at a 1.5% surfactant concentration (Table 2).

The concentration of the polymer provided a positive effect on particle size. Raising the polymer concentration to 3% *w*/*v* led to an increased size of particle. For example, formulations number 6 and 13, having a polymer concentration of 1% *w/v,* had particle sizes of 100 nm and 136 nm, respectively. On the other hand, formulations number 11 and 7 demonstrated particle sizes of 200 nm and 227 nm, respectively, with a 2% *w*/*v* polymer concentration. Again, increasing the polymer concentration (3% *w*/*v*) led to larger particle size: 240 nm.

On the other hand, sonication time demonstrated a negative impact on particle size. The formulations number 6 and 1 achieved particle sizes of 121 nm and 100 nm, respectively, with 12 min of sonication. With the same sonication time, formulation 16 exhibited a 232 nm particle size, probably due to a combined effect. Formulations 2 and 10 achieved a particle size of 230 nm and 170 nm, respectively, by sonication for 3 min.

### 2.3. Response 2: Effect on % Drug Release

The impact on % drug release of various excipients used in the formulation is explained by quadratic equation (Table 3).

In the above equation, the terms A, B, C, AB, AC, BC, A^2^, and B^2^ are significant. The Model F-value, 29.09, indicated a significant model for the % drug release. The lack-of-fit F-value of 0.53 entails insignificant for the model to fit. The polymer concentration positively impacted the % drug release. Formulation number 14 demonstrated that a 45% drug release had a 1% *w*/*v* polymer concentration. The increase of polymer concentration to 2% *w*/*v* led to an increased drug release: 71% and 75%, as seen in formulations number 3 and 17, respectively. Furthermore, after an increase in the polymer concentration of the formulation to 3% *w*/*v*, an increase in drug release was observed, such as cases of 89% and 83% in formulations number 1 and 10, respectively (Table 2).

### 2.4. Response 3: Effect on the PDI

The effect on the PDI of various excipients used in the formulation is explained by the quadratic equation (Table 3).

Surfactant concentration provided both positive and negative effects on the PDI. The observed PDI values were achieved: 0.123 and 0.231 at 1.5% and 2.5% of *w*/*v* surfactant concentration, corresponding to formulations number 1 and 5, respectively. Similarly, the polymer concentration provided both a positive and a negative effect on the PDI. When increasing the concentration of the polymer from 1 to 3% *w*/*v*, the PDI was initially increased and later decreased. For example, the polymer concentration of 3% *w/v,* as seen in formulations 4 and 5, indicated PDI values of 0.221 and 0.231, respectively. On the other hand, sonication time had a less negative impact on the PDI of formulations (Table 2).

In view of the above obtained outcomes, the optimized formulation was generated using a point-prediction technique with a Box–Behnken design. The optimized formula for preparation included a polymer concentration (2.94% *w*/*v*), surfactant concentration (2.20% *w*/*v*), and sonication time (11.39 min). The experimental design predicted a particle size of 92.76 nm, a % drug release of 89.31%, and a PDI of 0.102. The experimental or observed values of particle size, % drug release, and PDI were 95.35 ± 10.25 nm, 70.42 ± 7.25%, and 0.109, respectively.

### 2.5. Characterization of Dox–ErloNPs

#### 2.5.1. Particle Size and Zeta Potential

**Figure 3:**
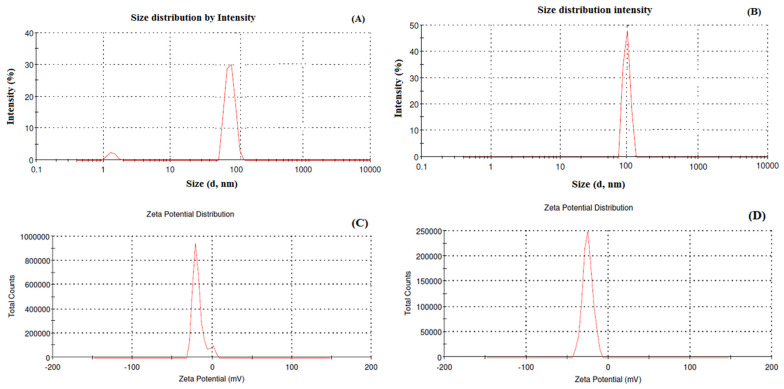
Particle size and size distribution analysis of Dox–Erlo NPs (**A**), particle size and size distribution analysis of Dox–Erlo-NP conjugates (**B**), zeta potential of Dox–Erlo NPs (**C**) and Dox–Erlo-NP conjugates (**D**).

**Figure 4:**
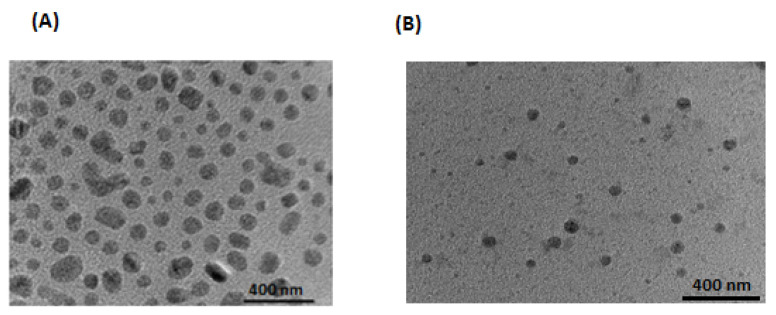
Transmission electron microscopic image of Dox–Erlo NPs (**A**), Dox–Erlo NPs conjugate (**B**).

The particle size and zeta potential of Dox–Erlo NPs and Dox–Erlo-NP conjugates are shown in Figure 3A,B. Dox–Erlo NPs demonstrated a particle size of 95.35 ± 10.23 nm. On the other hand, Dox–Erlo-NP conjugates appeared at a particle size of 110.12 ± 9.2 nm. The zeta potential of Dox–Erlo NPs and the Dox–Erlo-NP conjugates were −18.1 mV and −25.1 mV, respectively, as is shown in Figure 3C,D. The size range of the Dox–Erlo NPs and the Dox–Erlo-NP conjugates was between 50 and 150 nm (Figure 4A,B). The entrapment efficiency % of Erlo and Dox was 80 ± 2.3% and 78 ± 4.8%, respectively, and the polydispersity index (PDI) of the Dox–Erlo NP formulation was reported be 0.1027. The predicted NP size of the optimized formulation was 92.7661 nm, vs. an experimental particle size of 95.35 ± 10.25, reporting a percentage error of 2.79%. On the other hand, the % drug release of the optimized NPs was 89.91%, vs. the experimental value of 79.203 ± 0.24%, demonstrating a percentage error of 11.90%. The PDI of the predicted formulation was 0.102, compared to a PDI of 0.10, for the experimental value, demonstrating a percentage error of 6.8% (shown in Table 5).

#### 2.5.2. DSC of Dox–Erlo NPs

**Figure 5:**
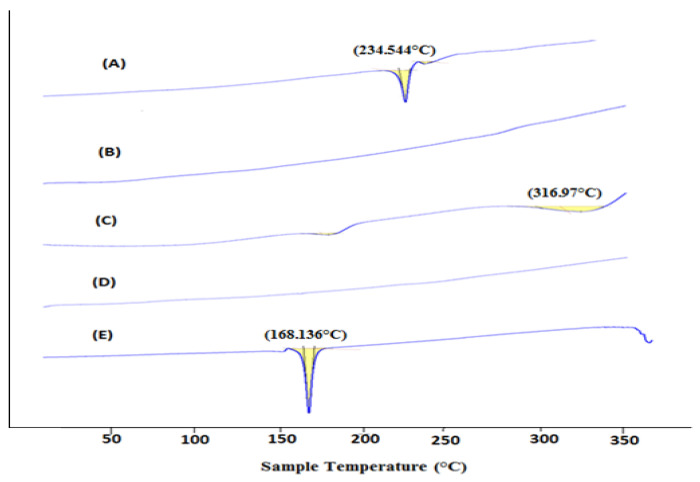
DSC thermogram of Erlo (A), cinnamon biopolymer (B), polyvinyl alcohol (C), Dox–Erlo NPs (D), and Dox–Erlo-NP conjugates (E).

DSC is used for the physicochemical characterization of the nature of substance. The DSC peaks of Erlo, cinnamon biopolymer, polyvinyl alcohol, Dox–Erlo NPs, and Dox–Erlo-NP conjugates are shown in Figure 5. The pure Erlo has a characteristic peak at 234.544 °C. Polyvinyl alcohol has shown a peak at 316.97 °C. Further, the endothermic peak, obtained at 168.136 °C, corresponds to the mannitol that was detected in the Dox–Erlo-NP conjugate in Figure 5.

#### 2.5.3. FT-IR Spectral Analysis

**Figure 6:**
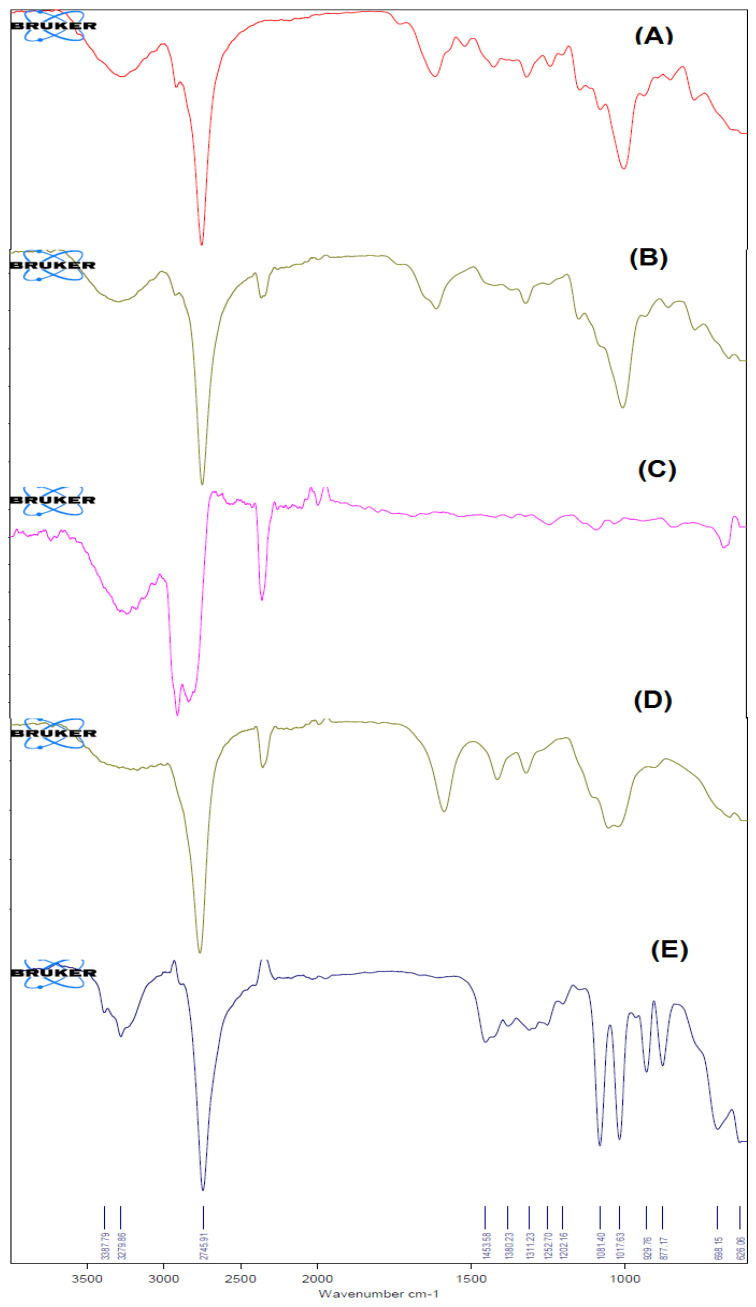
The FT–IR Spectra of Erlo (**A**), cinnamon polymer (**B**), polyvinyl alcohol (**C**), Dox–Erlo NPs (**D**), and Dox–Erlo NP conjugates (**E**).

FT-IR spectroscopy characterized the chemical stability of NPs encapsulated in the core of the biopolymer. The FT-IR spectra of Erlo, biopolymer, polyvinyl alcohol, Dox–Erlo NPs, and Dox–Erlo-NP conjugates are indicated in Figure 6. The structure of Erlo shows a 2-methoxy ethoxy group (C-O stretching) and amino-group (N-H stretching) of quinazoline ring. The biopolymer demonstrated a peak around 2743.12 cm^−1^, and 2918.33 cm^−1^ belongs to the carboxylic acid group. The Erlo drug demonstrated absorption bands at 3267.14 cm^−1^, corresponding to N-H stretching, and at 1081.18 cm^−1^, attributed to C-O stretching (Figure 6).

#### 2.5.4. Proton Nuclear Magnetic Resonance (^1^H NMR)

**Figure 7:**
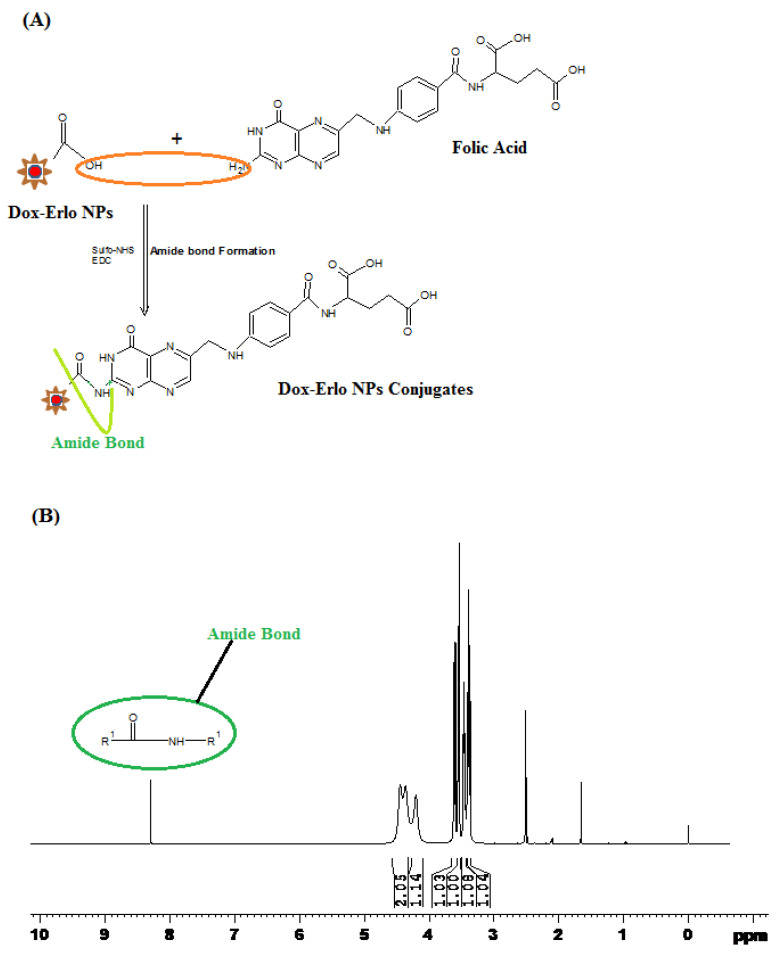
Schematic diagram of amide bond formation of Dox–Erlo NPs (**A**) and the^1^H NMR spectrum of Dox–Erlo-NP conjugates (**B**).

The formation of an amide bond between the primary amine group (-NH2) of folic acid and the carboxylic acid group from the Dox–Erlo NPs through a conjugation reaction is shown in Figure 7A. The ^1^H NMR spectroscopy of the amide linkage formation in Dox–Erlo-NP conjugates is shown in Figure 7A,B. An appearance of the signals at 8.3921 ppm indicated the formation of an amide bond through a reaction between the activated ester group of the polymeric nanoparticles and the primary amine group of folic acid (Figure 7B).

#### 2.5.5. X-ray Diffraction Analysis

**Figure 8:**
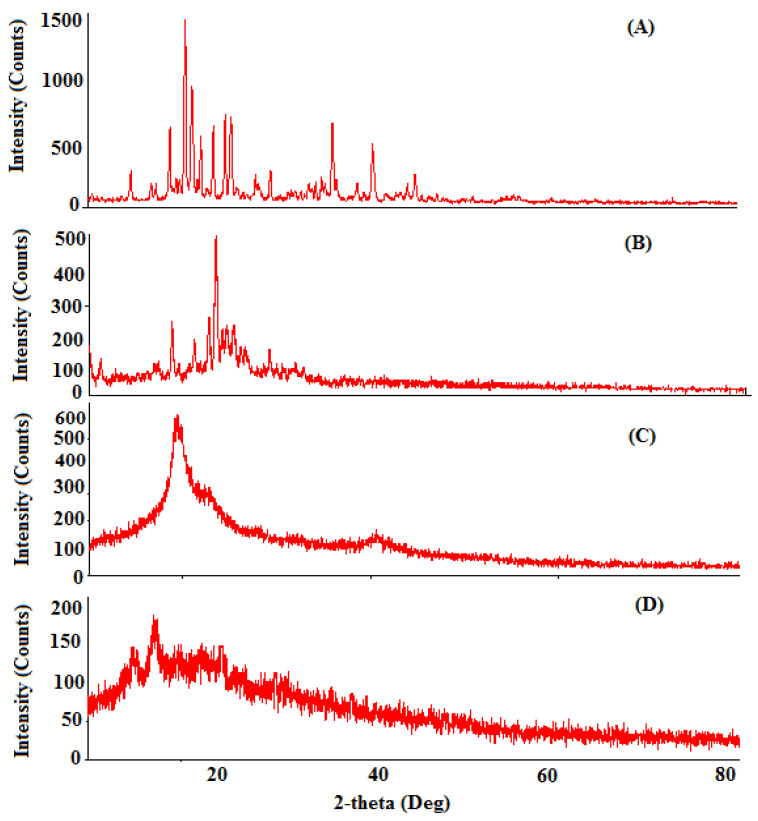
The XRD diffraction analyses of Erlo (**A**), cinnamon biopolymer (**B**), Dox–Erlo NPs (**C**), and Dox–Erlo-NP conjugates (**D**).

The confirmation of the physicochemical drug behavior encapsulation in the NPs was further illustrated with help of X-ray analysis. The X-ray diffraction patterns of Erlo, cinnamon biopolymer, Dox–Erlo NPs, and Dox–Erlo-NP conjugates are shown in Figure 8. The high-intensity characteristic peaks in the Erlo were observed at 2θ angles of 18.74°, 20.38°, 21.07°, 25.26°, 36.04°, and 40.33°,indicating their crystalline nature (Figure 8A).The low-intensity peaks in the biopolymer were observed at 2θ angles of18.84°, 22.72°, 23.48°, and 25.41°. Moreover, the peaks prevailed in the biopolymer, as is shown in Figure 8B, suggesting a less-crystalline nature. However, the peaks of crystalline nature were produced at a very low intensity or disappeared in the diffraction patterns of Dox–Erlo NPs and the Dox–Erlo conjugates, indicating that Erlo and Dox were in amorphous or molecular states in the NPs.

#### 2.5.6. In Vitro Drug Release

**Figure 9:**
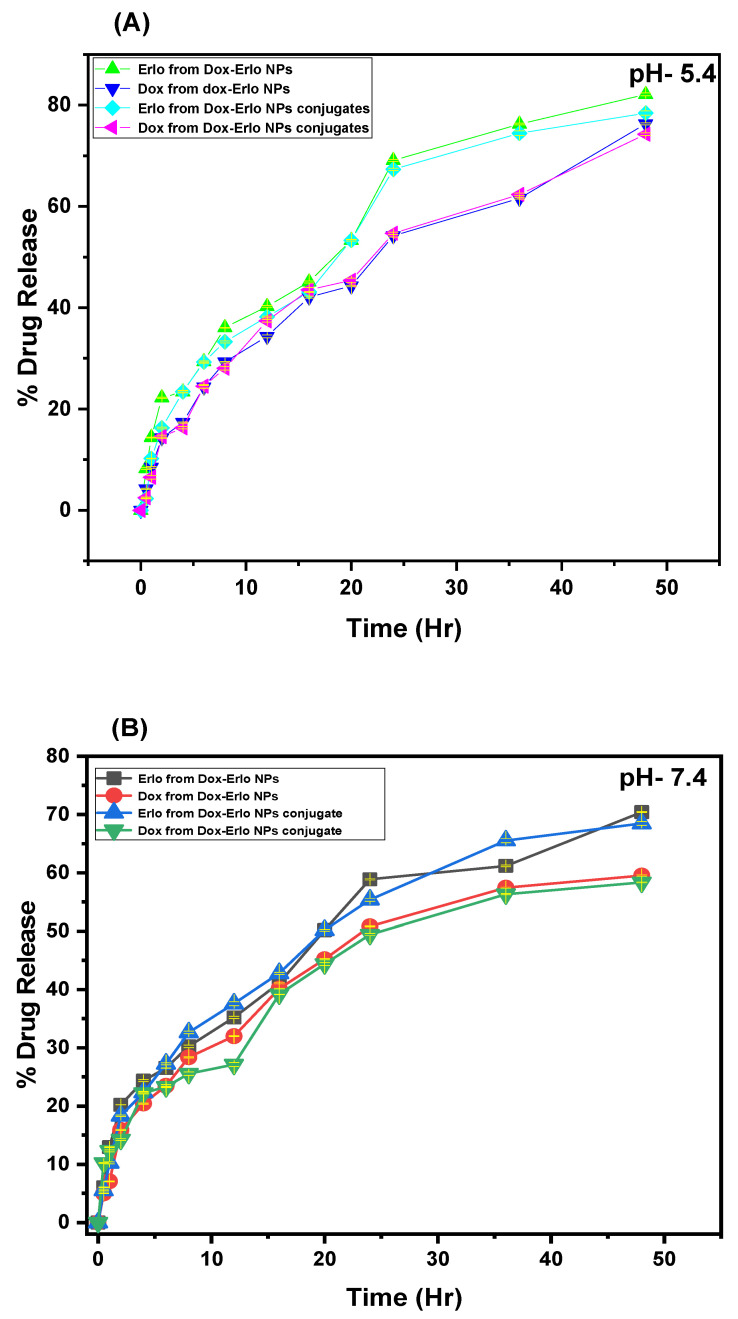
In vitro drug releases of Erlo and Dox from the plain drug, Dox–Erlo NPs, and Dox–Erlo-NP conjugates at a pH of 5.4 (**A**) and a pH of 7.4 (**B**).

In vitro release studies were performed for Erlo and Dox from Dox–Erlo NPs and Dox–Erlo-NP conjugates at a pH of 7.4 (simulating a physiological pH) and a pH of 5.4 (mimicking the pH of acidic, intracellular, endosomal cancer cells), respectively. The maximum amounts of Dox released from the Dox–Erlo NPs and Dox–Erlo-NP conjugates at a pH of 7.4 were 59.54 ± 0.10% and 58.34 ± 0.073%, respectively. On the other hand, at a pH of 5.4, the maximum amounts of Dox release from the Dox–Erlo NPs and Dox–Erlo-NP conjugates were 76.29 ± 0.19% and 74.24 ± 0.24%, respectively. The amounts of Erlo released from Dox–Erlo NPs and Dox–Erlo-NP conjugates at a pH of 7.4 were 70.42 ± 0.05% and 68.47 ± 0.29%, respectively. Similarly, at a pH of 5.4, the amounts of Erlo released at the end of 47 h were 82.11 ± 0.30% and 78.43 ± 0.39%, respectively, as is shown in Figure 9A,B.

#### 2.5.7. Kinetic Release Model

The releases of Erlo and Dox from Dox–Erlo NPs and their conjugates were fitted to the different release kinetic model. The exponent (*n*) expressed aFickian or non-Fickian pattern of drug release. The exponent value for the zero-order release/Case II transport, *n* = 1; non-Fickian diffusion, 0.5 < *n* < 1; or relaxational release, *n* > 1 is considered. The model of good fit was judged based on the regression coefficient value (R^2^). The regression coefficient (R^2^) values for such models were determined, for example: zero order (R^2^ = 0.9002), first order (R^2^ = 0.9744), Higuchi (R^2^ = 0.9096), Korsmeyer–Peppas (R^2^ = 0.9793), and Hixson–Crowell (R^2^ = 0.9593) were estimated. It was observed that Korsmeyer–Peppas showed a good fit to the model of (R^2^ = 0.9793) for the Erlo release from Dox–Erlo-NP conjugates at apH of5.4. The *n*-value was 0.4805 and the k-value was 3.0543. The release of Dox from Dox–Erlo-NP conjugates at a pH of 5.4 fitted in a different kinetic model, and the regression coefficients for various kinetic models were provided: zero order (R^2^ = 0.8541), First order (R^2^ = 0.9231), Higuchi matrix (R^2^ = 0.9233), Korsmeyer–Peppas (R^2^ = 0.9751), and Hixson–Crowell (R^2^ =0.9025) [Table 6]. The Korsmeyer–Peppas was the best-fitted model, with a regression value of (R^2^ = 0.9751), an *n*-value of 0.5951, and a k-value of 2.4551. The release mechanism indicated an anomalous, non-Fickian diffusion of Dox, both via diffusion and biopolymeric matrix erosion; on the other hand, Erlo was released via biopolymeric matrix erosion.

In the determination of the Erlo release from the Dox–Erlo-NP conjugates at a pH of 7.4, the regression coefficient values for the zero order (R^2^ = 0.8704), first order (R^2^ = 0.9537), Higuchi matrix (R^2^ = 0.9190), Korsmeyer–Peppas (R^2^ = 0.9782), and Hixson–Crowell (R^2^ = 0.9306) were determined. Among these models, Korsmeyer–Peppas demonstrated the highest regression value (R^2^ = 0.9782), with a release exponent *n*-value of 0.5294 and a k-value of 2.733 selected. Further, the regression coefficients for Dox release from Dox–Erlo-NP conjugates at a pH of 7.4 were determined using the same models: zero order (R^2^ = 0.8718), first order (R^2^ = 0.9294), Higuchi (R^2^ = 0.9062), Korsmeyer–Peppas (R^2^ = 0.9709), and Hixson–Crowell (R^2^ =0.9125). Due to the emergence of a highest regression coefficient value for the Korsmeyer–Peppas model, it was selected as the model of good fit. It indicated an *n*-value of 0.41 and a k-value of 2.9451 [Table 7]. The mechanism of drug release expressed that Dox was released via Fickian diffusion following both diffusion and biopolymeric matrix erosion. On the other hand, the Erlo release mechanism followed an anomalous or non-Fickian diffusion through biopolymeric matrix erosion [27].

#### 2.5.8. Hemolysis Study

Hemolysis experiments were carried out to ensure the biocompatibility of the in-house-built NPs and NP conjugates in the bloodstream and to obtain information about the charge–particle interaction with biomolecules in terms of thrombosis and hemolysis in vivo. These interactions enable damage to erythrocytes and thereby acquit hemoglobin from erythrocytes. It was observed that increasing the NP doses led to an increased release of hemoglobin from the erythrocytes. The hemolytic analysis revealed that RBC damages were less than 6–8% in any of the concentrations (1.5 mg, 3 mg, and 6 mg) used in the experiment relating to placebo NPs, Dox–Erlo NPs, and Dox–Erlo-NP conjugates.

#### 2.5.9. Cytotoxicity Assay

**Figure 10:**
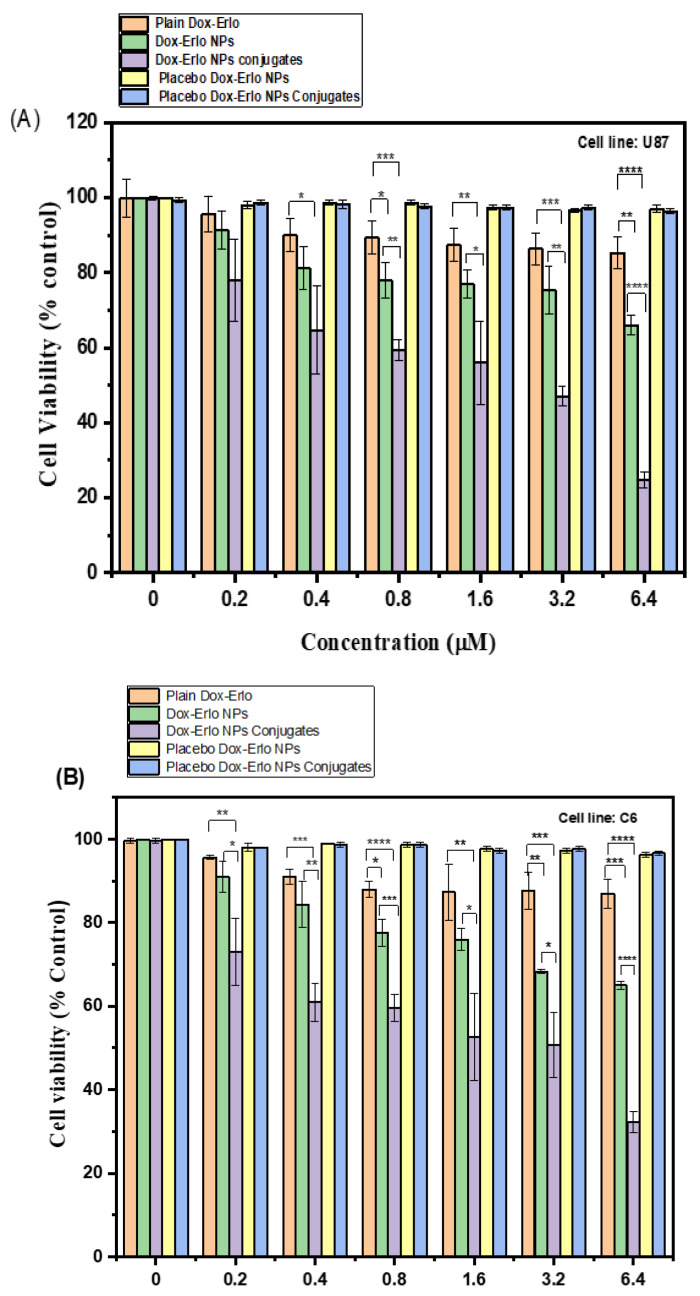
The percentage cell viability following 24 h of treatment with various doses of plain Dox–Erlo, Dox–ErloNPs, Dox–Erlo-NP conjugates, placebo Dox–Erlo NPs, and placebo Dox–Erlo-NP conjugates on U87 (**A**) and C6 (**B**) glioma cell lines. The experiments were performed in triplicate with mean ± S.D (*n* = 3). Significance value * (*p <* 0.05), ** (*p <* 0.01), *** (*p <* 0.001), **** (*p <* 0.0001) relative to pure Dox-Erlo.

The results of the MTT assay analysis of plain drugs, Dox–Erlo-NPs, and Dox–Erlo-NP conjugates on glioma cell lines (U87 and C6) at varying concentrations (0.20 µM, 0.40 µM, 0.80 µM, 1.6 µM, 3.2 µM, and 6.4 µM) are shown in Figure 10A,B. The Dox–Erlo-NP conjugates significantly depleted the count of viable cells to 24.66 ± 2.08% when compared to Dox–ErloNPs (66 ± 2.6%) and plain drugs (85.33 ± 5.5%) in glioma U87 cells. Oppositely, Dox–Erlo-NP conjugates reduced the viable cell count to 32.33 ± 2.51% when compared to Dox–ErloNPs (65 ± 1%) and plain drugs (87 ± 3.46%) in glioma C6 cells. Furthermore, cell death was expressed in terms of the IC50 related to the dose of drug, which killed 50% of cancer cells in a specified time period, i.e., the inhibitory concentration (IC50). The IC50 values of plain Dox–Erlo, Dox–ErloNPs, and Dox–Erlo-NP conjugates were 26.589 µM, 9.830 µM, and 3.064 µM, respectively, after 24 h in the U87 cell line. The IC50 values of plain Dox–Erlo, Dox–ErloNPs, and Dox–Erlo-NP conjugates were determined to be 32.60 µM, 8.625 µM, and 3.350 µM, respectively, after 24 h in the C6 glioma cell line, shown in Figure 10A,B.

#### 2.5.10. Biodistribution Study

**Figure 11:**
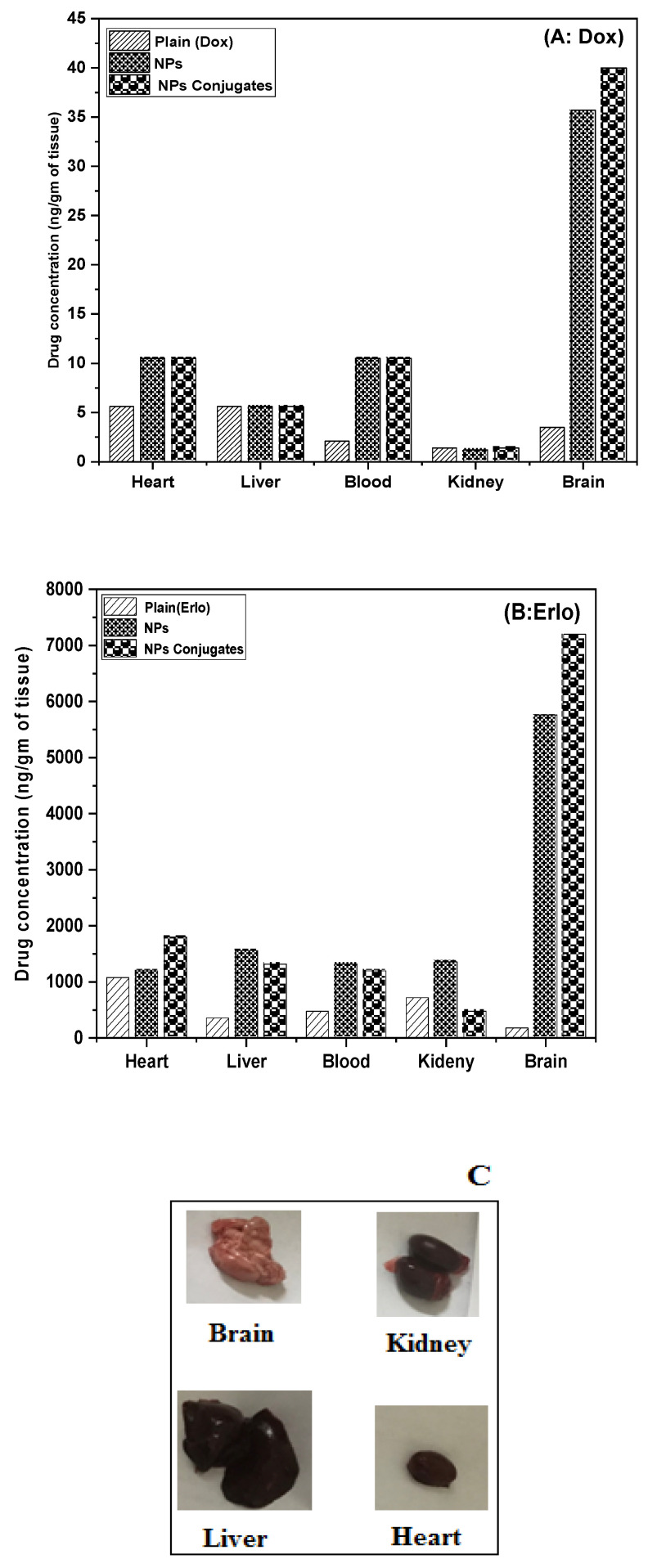
Graph representing the biodistribution of Dox (**A**) and Erlo (**B**) from plain, Dox–Erlo NPs, and Dox–Erlo-NP conjugates. Isolated organs of animals after 24 h of dose (**C**).

The tissue homogenates from various organs such as the liver, kidney, brain, and blood of rats were extracted and analyzed via HPLC for the presence of Dox and Erlo. It was found that a significant amount of Dox and Erlo were estimated in the brain as compared to drug suspension (*p* < 0.05). The biodistribution studies of the formulation in various organs are expressed in Figure 11.

#### 2.5.11. Stability Study

The stability study was performed as per guideline issues under a stability study [28]. The Dox–Erlo-NP conjugates’ stability experiments under a specific set of conditions are expressed in Table 8. The particle size observed was 109.45 ± 12.48 nm at 25 ± 2 °C, 65 ± 5% RH at the end of 90 days. However, at an elevated temperature of 40 ± 2 °C, 75 ± 5% RH, a particle size of 115.33 ± 12.38 nm was observed. Similarly, the surface charge on the Dox–Erlo-NP conjugates at temperatures of 25 ± 2 °C, 65 ± 5% RH and 40 ± 2°C, 75 ± 5% RH were recorded as −21.1 ± 4.01 and −20.4 ± 3.20 mV, respectively. The entrapment efficiencies after a stability period of 90 days were calculated at 76 ± 5.3% and 73 ± 3.3%, respectively.

## 3. Discussion

The treatment of a glioma is impeded via the invasiveness or the inadequacy of drugs penetrating the BBB [29]. The current study was designed to develop, characterize, and evaluate Dox–Erlo NPs and folate-armored Dox–Erlo-NP conjugates for targeting glioma cancer via a nose-to-brain route. The study aimed to improve the targeted specificity and promote the penetration of NPs to glioma cells to achieve the desired therapeutic concentration.

This biopolymeric, nanocarrier-based drug delivery is a novel approach for drug targeting to a specific region as it offers biodegradability and biocompatibility and is non-toxic to the vital organs of the body, as was disclosed in the hemocompatibility study. The folate-armored, polymeric nanocarrier has shown better biodistribution in the brain due to its higher permeability and penetration of the BBB. The developed biopolymer nanoconjugates were effective in glioma therapy as they enabled a controlled drug release over a prolonged time and a tunable size, by which they could approach the target domain, minimize off-target effects, and increase bio-stability. The TEM studies of the nanoconjugate were well-dispersed, uniform, de-aggregated, and consistent in size. The low PDI value showed that the developed preparations were consistent, homogeneous, and had a narrow size distribution. The zeta potential value indicated a negative surface charge on the nanoparticle formulation; the nanoparticles showed no agglomeration due to a same-charge surface repellence of each other, creating a resistive force that led to the enhanced stability of the nanosize system [30].

It has been proven that the over-expressed folate receptor on the tumor cells’ surface could be a specific target site for delivering cytotoxic agents [31]. In our study, the conjugation of folic acid to Erlo–Dox preparations was found to be at a higher concentration in the brain when compared to a non-conjugated preparation. This substantiates the higher efficacy of folate-conjugated nanoparticles when compared to plain NPs. The conjugated NPs’ formulation exhibited a remarkable cell death and higher concentration in the brain when compared to the unconjugated NPs, consistent with the previous literature. The conjugation of folate with NPs was confirmed by ^1^H NMR analysis. The results clearly indicated that conjugated NPs can be a promising, tumor-targeting carrier candidate. No endothermic peak in DSC was detected for the drug in Dox–Erlo NPs and conjugated NPs, suggesting that drug has been incorporated in the NPs. The DSC chromatogram of mannitol was detected in the Dox–Erlo-NP conjugates [32].

The functional peaks of the drug in FT-IR becoming flattened in the Dox–Erlo NPs and Dox–Erlo-NP conjugates indicated that the drug was encapsulated in the biopolymeric core [33]. ^1^H NMR evidently revealed the conjugation of the primary amine group of folic acid with the carboxylic acid group of the polymeric NPs. In ^1^H NMR, the appearance of the signals at 8.3921 ppm indicated the formation of an amide bond by a reaction between the activated ester group of the polymeric nanoparticles and the primary amine group of the folic acid. Dox–Gefit-NP conjugates were synthesized, as indicated by the formation of amide bond. The appearance of this peak confirmed the conjugation of folic acid [34].

The prepared nanoparticles were nano-sized, having a desirable diameter of 95.35 ± 10.25 nm and 110.12 ± 9.2 nm for the NPs and conjugates, respectively, and exhibited a sustained release of the drug under physiological conditions [35,36].The zeta potential value indicated a negative surface charge on the nanoparticle formulation and no non-agglomerated NPs, probably due to the same-charge surface repellence of each other, with the resultant resistive force leading to an enhanced stability of the nanosize system [37]. Furthermore, the mean PDI of the NPs in our study was 0.109, showing that the developed preparations were consistent, homogeneous, had a narrow particle-size distribution, were monodispersed, and were satisfactory [38,39].

Free Dox and Erlo can cause brain toxicity, cardiotoxicity, and kidney or liver damage. In this study, converting them to NPs and encapsulating them within a biopolymer helped to prevent the toxic side effects of systemic Dox and Erlo administration. The encapsulation of the drug was confirmed by DSC, with FTIR analysis as standard practice. It is being proven that biopolymers demonstrate non-toxicity and short immunogenicity, are bio-absorbable, and have subsequently good biocompatibility. Hence, their use can minimize the potential hazards of cytotoxicity. In the present study, a *Cinnamomum zeylanicum* biopolymer was extracted and used as a nanoparticle-carrier material to achieve a higher concentration of the drug at the targeted tumor site with reduced toxicity. The cytotoxicity study result showed no toxicity of the biopolymer, signifying that the biopolymer is safe and biocompatible [40].

The results of the % drug release assessment demonstrated that Erlo released faster than Dox from NPs. During the initial phase of drug release, an abrupt release was demonstrated, followed a controlled release for a long time. This may be due to Erlo becoming entrapped in the exterior layer, while Dox was encapsulated in the interior core of the NPs [41]. Further, it was observed that release of Erlo and Dox was found to be higher at an acidic, intracellular, endosomal pH of 5.4 when compared to a pH of 7.4. It is worthwhile to disclose herein that the microenvironment of a tumor is slightly more acidic than the physiological fluid [42]. The higher drug release at an endosomal pH of 5.4 in the slightly acidic microenvironment of the tumor may be attributed to the fact that the protonation of the biopolymer and drugs resulted in a higher dissolution of Dox and Erlo from the internal polymeric complex of the NPs in the acidic environment. The pH-dependent drug release is highly desirable for cancer-tissue targeting and also minimizes non-selective drug release in systemic circulation. It also provides sufficient drug concentration upon cellular internalization, which is mediated via endosomal escape and lysosomal fusion [43,44,45]. After fitting the drug-release data in kinetic models, the exponent value *n* of Erlo from Dox–Erlo NPs at a pH of 5.4 and a pH of 7.4 and Dox from Dox–Erlo NPs at a pH of 5.4 showed that the release mechanism was diffusion (non-Fickian). However, Dox at a pH of 7.4 demonstrated a Fickian drug release mechanism. The findings indicate that Erlo and Dox release from Dox–Erlo-NP conjugates was ascertained via diffusion from polymeric core. The hemolysis assay disclosed that the maximum concentration of formulation was 6 mg; when tested for hemocompatibility, this did not cause significant hemolysis. The hemolysis study was resembled preceding work in the literature [46,47]. The developed formulations were conceived to be as least toxic or non-toxic and are regarded as safe and hemocompatible for in vivo administration. As per the experimental observation, the stability of the Dox–Erlo-NP conjugates were maintained, as indicated by an insignificant alteration in particle size, zeta potential, and entrapment efficiency after an analysis of the sample at fixed intervals of time during a storage period of 90 days (*p* > 0.05). This further indicates that in-house-built Dox–Erlo NPs were robust and consistently in line with the ICH stability-testing guidelines [48,49].

The efficacy of the formulation was studied by assessing the IC50 values and the percent of depletion of cancer cells [50]. The cell-killing potency of the formulations was dose and time-dependent. The MTT assay interpreted that the Dox–Erlo-NP conjugates successfully decreased the % cell viability according to the concentration of the drug in NPs and the drug delivery into the cells [51]. The existing literature demonstrates that using a synergistic combination of EGFR inhibitor viz., Erlotinib provides the cells susceptible to apoptosis with exposure to the DNA-destructive agent doxorubicin [52].

The analytical estimation showed that drug concentration was achieved in the vital organs (the heart, liver, and kidney) with small quantities of Dox and Erlo when compared to the targeted brain, which may be attributed to the partitioning behavior of the nanosized Dox–Erlo NPs and the Dox–Erlo-NP conjugates via endothelial fenestration. Overall, the concentrations of Dox and Erlo achieved in the target organ, i.e., in the brain, were significantly higher than in other organs of the body (*p* < 0.05), indicating the specific delivery of the formulated conjugate in the targeted region of glioma cancer [53].

## 4. Material and Methods

### 4.1. Materials

Erlotinib (Mol wt = 393.436, purity of ≥95%) was a gift sample from Natco Pharma Ltd. UPSIDC (Dehradun, India). Doxorubicin also a gift sample from Neon Laboratories Pvt. Ltd. (Ghaziabad, India).The cinnamon biopolymer was purchased from Shree Ram Overseas (New Delhi, India). The polyvinyl alcohol (PVA) was received from Sisco Research Laboratory Pvt. Ltd. (Mumbai, India). The cross-linking agents EDC [1-(3 Dimethylaminopropyl)-3-Ethyl Carbodiimide Hydrochloride] and Sulpho-NHS [N-Hydroxysuccinimide] were received from Sisco Research Laboratories Pvt. Ltd. (Mumbai, India). The solvent, Dimethyl Sulfoxide (DMSO), was obtained from Merck Pvt. Ltd. (Mumbai, India), and acetone was obtained from SD Fine Chem Pvt. Ltd. (Mumbai, India), HPLC-grade water and other reagents were used as received.

### 4.2. Cytotoxicity Study

#### Materials

The specified materials for the study of cytotoxicity, such as culture media, penicillin streptomycin, MTT (4, five-dimethylthiazol-2yl)-2, five-diphenyl tetrazolium bromide), fetal bovine serum (FBS), and Dulbecco’s Modified Eagle Medium (DMEM) were bought from Himedia (Mumbai, India). The phosphate-buffered saline (PBS) was purchased from (Himedia, India). The cell lines C6 and U87 were received from NCCS, Pune, India. Cells were stored at 37 °C and 5% CO_2_ in a humidified CO_2_ incubator to maintain continuous growth.

### 4.3. Formulation Optimization Using Statistical Design

The optimization of formulation was carried out through Design-Expert Software (Design-Expert version 12, State-Ease^®^ Inc., Minneapolis, MN, USA) using Box–Behnken design (BBD).The expert design used a three-level, three-factor BBD which produced seventeen experimental runs for optimizing the formulation. The investigative impact of independent variables, viz., (A) polymer concentration; (B) surfactant concentration; and (C) sonication time on thefactors (R1) particle size (nm); (R2) PDI, and (R3) drug release (%) were studied. The levels of independent variables under study were used as low (−1), intermediate (0), and high (+1), and their impact on the responses R1, R2, and R3 are shown in Table 1. This design comprehensively explained the major, combined, and quadratic effect of factors A, B, and C on various selected responses in the study of the formulation. The optimization of formulation based on Design-Expert version 12, State-Ease^®^ Inc. (Minneapolis, MN, USA) was reported in various preceding works [54].

### 4.4. Preparation of Dox–Erlo-Loaded NPs

**Figure 12:**
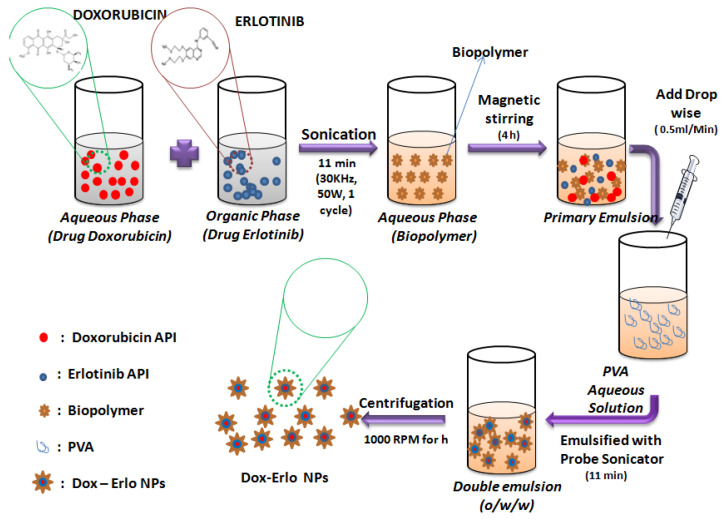
Schematic representation of the preparation of Dox–Erlo NPs by double-emulsion evaporation method.

Dox–Erlo-loaded biopolymeric NPs were developed by implementing a modified, double-emulsion solvent-evaporation technique [55]. The technique involved the preparation of an Erlo solution in an organic phase (1 mg/mL), a Dox solution in an aqueous phase (5 µg/mL), a biopolymer in an aqueous phase (29.4 mg/mL), and the preparation of an aqueous PVA solution. Primarily, the solutions of Erlo (1 mg/mL) and Dox (5 µg/mL) were transferred slowly using an injectable needle in the aqueous biopolymer solution (2.94% *w*/*v*) and emulsified slowly using a probe sonicator (Hielscher ultrasonicator, Berlin, Germany) (02 min, 30 KHz power, 50 W, 01 cycle) to obtain a polymeric core of the drug as a primary emulsion (*o/w*). Second, this primary emulsion was transferred into the aqueous PVA solution (2.20 % *w*/*v*) slowly, using an injection needle at a rate of 0.5 mL/min. This was then emulsified for 11 min with the probe sonicator (30 KHz power, 80 W, 01 cycle) to obtain a secondary emulsion comprising a nanoparticle suspension. Thereafter, the preparation was stirred magnetically at 1000× *g* rpm for 4 h at ambient temperature to allow for the evaporation of the organic phase. Further, NPs were held open overnight to obtain hard and dry particles. The nanoparticles were then ultracentrifuged at 15,000× *g* rpm (OptimaTM LE-80K Ultracentrifuge) for 30 min and washed (*n* = 3) to free the NPs of un-entrapped drug and free biopolymer matter. The Dox–Erlo-loaded nanoparticle was then re-dispersed in water and lyophilized to dryness for future characterization. The NP preparation steps are illustrated in Figure 12.

### 4.5. Surface Modification of Dox–Erlo Biopolymeric NPs

**Figure 13:**
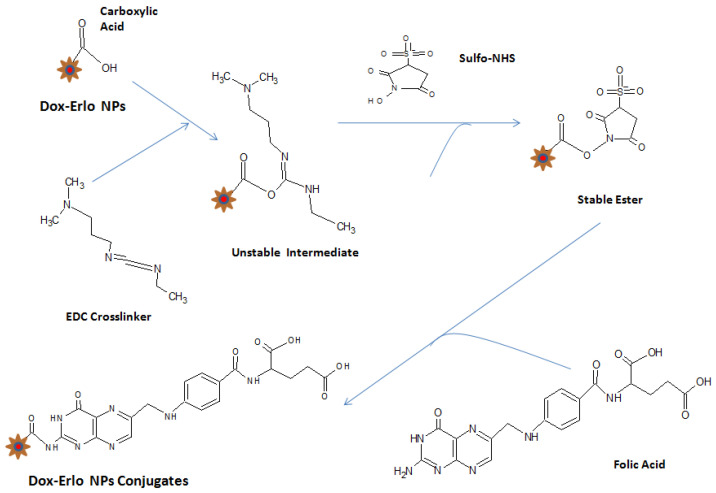
Surface modification of Dox–Erlo NPs.

The nanoparticles were re-dispersed to 10 mg/mLin double-distilled water and incubated with 0.1% of 1-(3 Dimethylaminopropyl)-3-Ethyl Carbodiimide Hydrochloride (EDC. HCl) and N-hydroxysuccinimide (sulpho-NHS, 0.05% *w*/*v*), for 5 h in a biological shaker to activate the carboxylic group. In the course of the first step of the coupling reaction, an unstable intermediate was formed on reaction with the EDC cross-linker, which further reacted with sulpho-NHS and formed a stable ester. After incubation, the amine-reactive stable ester (sulpho-NHS NPs) was washed three times with distilled water. In the consequent step, the stable ester (sulpho-NHS NPs) was re-dispersed with folic acid (0.1% *w*/*v*) and incubated overnight to hasten the coupling reaction at ambient temperature in an end-to-end biological shaker. The folate-conjugated Dox–Erlo NPs were subjected to centrifugation for half an hour at 15,000× *g* rpm; thereafter, the supernatant was withdrawn and washed to remove traces of un-conjugated EDC and sulpho-NHS. The conjugated Dox–Erlo NPs were dried via lyophilization for further use. The surface-modification steps of the NPs are shown in Figure 13.

### 4.6. Characterization of Dox–ErloNanoparticles

#### 4.6.1. Particle Analysis and Z-Average

The distribution of particles and the Z-average of Dox–Erlo NPs were analyzed by utilizing a Zetasizer 1000 HS (Malvern Instruments, Worcestershire, UK). As per the standard procedure, the Dox–Erlo NPs were re-distributed in HPLC-grade water (0.5 mg/mL) and sonicated for one minute for one cycle at 60 Hz. The sizing analyses were computed and recorded three times (*n* = 3).

#### 4.6.2. Drug Entrapment and Loading in NPs

Erlo and Dox entrapment in the Dox–Erlo NPs was evaluated by the centrifugation of the formulation at an elevated speed of 15,000× *g* rpm at 4 °C for 30 min (C24, REMI Refrigerated Centrifuge, Mumbai, India). The amount of un-incorporated drug was estimated by reading the absorbance of the supernatant at 342 nm and 480 nm using a UV-visible spectrophotomer.

The % entrapment efficiency and the loading of drug were estimated using following equation:

(1) $$\%\;Entrapment\;efficiency=\frac{Total\;amount\;of\;drug-amount\;of\;drug\;in\;the\;supernatant}{Total\;amount\;of\;drug}\times 100$$

#### 4.6.3. High-Resolution Transmission Electron Microscopy (HR-TEM)

The morphological characterization of the nanoparticles was studied using a JEOL, JEM 2100 Plus, (Japan) operated at 80 to 200 kV at an ultra-high resolution (UHR). The re-dispersed nanoparticles (0.5 mg/mL) were sonicated for 1 min by dispersion in water. Further, one drop of nanoparticles was stretched over a permeable film grid and dried for ten minutes. Microscopic images were observed and captured at 80 to 200 kV.

#### 4.6.4. Fourier Transform Infrared Spectroscopy (FT-IR)

The FT-IR spectra of Erlo, biopolymer, PVA, Dox–Erlo NPs, and Dox–Erlo-NP conjugates were characterized by FT-IR (Tensor 37, Bruker, MA, USA). Sample of weights of 5 mg were directly placed into the light-beam path and spectra were recorded in a scanning range of 4000–400 cm^−1^.

#### 4.6.5. Differential Scanning Calorimetry (DSC)

The DSC technique was used to compute the melting point and physical state of the drugs Dox, Erlo, biopolymer, PVA, Dox–Erlo NPs, and Dox–Erlo-NP conjugates by using DSC (Pyris 4 DSC, Perkin Elmer, Waltham, MA, USA). This technique estimated the difference in temperature between a test sample and a reference as a function of the time and temperature when the samples underwent temperature scanning in a range of 50–350 °C in a controlled atmosphere.

#### 4.6.6. X-ray Diffraction (XRD)

XRD analyses of Dox, Erlo, biopolymer, PVA, Dox–Erlo NPs, and Dox–Erlo-NP conjugates were characterized by a PAN analytical X’pert PRO, (Netherland) working at 40 kV, 30 mA, and 2-theta angle ranges (0° to 80°) using monochromatic CuKa-radiation (k = 1.5406 Å).

#### 4.6.7. Proton-Nucleic Magnetic Resonance (^1^H-NMR)

The ^1^H-NMR spectra of Dox–Erlo NPs and Dox–Erlo-NP conjugates were acquired on a Bruker Avance—II (Terre Haute, IN, USA) at 400 MHz. The chemical shifting was reported in ppm for the structure elucidation of Dox–Erlo-NP conjugates, which were compared with the Dox–Erlo NPs to confirm the conjugation by using a DMSO solvent and investigating the surface chemistry of the nano-conjugate [56].

#### 4.6.8. In Vitro Release Studies

In vitro Dox and Erlo release of the developed formulations of Dox–Erlo NPs and Dox–Erlo-NP conjugates were assessed by diluting the nanoparticles in PBS at a physiological pH of 7.4 and at an acidic, intracellular, endosomal pH of 5.4. The encapsulated drug NPs were kept enclosed in a dialysis bag (Mol. wt cut-off = 60–8 kDa) with the ends tightened. The dialysis bag was then immersed in 50 mL of PBS at a pH of 7.4 and maintained at 37 ± 0.5 °C with a gentle shaking at 50 rpm. A sample (1 mL) was adjourned at programmed intervals and replaced with an equal volume of fresh PBS at a pH of 7.4 and a pH of 5.4. Samples were examined using UV-visible light with wavelengths of 342 nm and 480 nm.

#### 4.6.9. Hemolysis Study

The hemolysis study was carried out by collecting blood from adult rats in EDTA-coated tubes, followed by centrifugation at 2000 rpm for 10 min to separate the cells and plasma. Further, the sediment cells were washed (*n* = 3) with PBS at a pH of 7.4. Various concentrations of NPs (placebo NPs, Dox–Erlo NPs, and Dox–Erlo-NP conjugates), including 1.5 mg, 3 mg, and 6 mg, were incubated with RBCs of number 1.5 × 10^7^ at 37 °C for 1 h. The samples were subsequently subjected to centrifugation for ten minutes at 2000 rpm. The supernatant was analyzed at 540 nm using UV-visible spectroscopy. An RBC hemolysis of 100% with Triton X-100 was considered to be a positive control and an RBC hemolysis of 0% after treatment with PBS was considered to be a negative control [57]. If the hemolysis was less than 10%, it was regarded as non-toxic. The following formula was used to calculate the hemolysis %:

% Hemolysis = [(Abs(treatment) − Abs(PBS))/Abs(Triton × −100)] × 100(2)

#### 4.6.10. Cytotoxicity Study

The test for the efficacy of the nanosystem composition in terms of therapeutic accumulation and internalization was investigated in cell lines C6 (ATCC, CCL107) and U87 (ATCC, HTB14) of glioma tumors in vitro. The cell-viability study of Dox–Erlo NP and Dox–Erlo-NP conjugate nanosystem was carried out using an MTT assay. The culture cells were treated with DMSO, and the formazan reagent formed (solubilized) was estimated using a spectrophotometer. MTT acts only on biologically active cells, and the activity of cells indicates the cells’ viability [58]. In this method, cell lines were added to a 96-well plate (106 cells/well) containing DMEM media and then incubated overnight at 37 °C in a humidified atmosphere in which the air was enriched with (5% *v/v*) CO_2_ tofacilitate the attachment of cells to the bottom of each well. Upon the well attachment of cultured cells, cells were treated with concentrations (between 0.20 and 6.4 µM) of Dox–Erlo-NPs and Dox–Erlo-NP conjugates and incubated for 24 h.

After the completion of the treatment, the media were removed carefully and incubation was repeated with 10 mL of MTT for 3 h. After the completion of the incubation, the optical density was measured at 570 nm in a microplate reader. Each experiment was performed in triplicate (*n* = 3). Untreated cells were related to the control group (100% cell viability), and the IC50 of the cells was determined. IC50 is the drug concentration that slows cell growth by 50% when compared to a control. It is calculated using a regression analysis of cell-viability studies.

Cell viability (%) was expressed as the mean viability (%) ± standard deviation (SD) (*n* = 3) using the following formula:

The cell viability (%) was represented as mean ± SD (*n* = 3) using following formula;

Percent cell viability = OD treated/OD controlled × 100(3)

#### 4.6.11. Biodistribution Studies

Animals were procured from an animal house prior to the experiment and maintained in polymeric cage as per animal ethical guidelines. The animals were housed at room temperature and exposed to 12 h of light/dark. They were kept on food and water ad libitum. Institutional Animal Ethics Committee (IAEC) guidelines were followed in conducting animal experiments as per the guidelines by DIT University, Dehradun, Uttarakhand, India (Ref no. DITU/IAEC/21-22/07-05). To investigate biodistribution, the administration of a single dose of a formulation such as pure Erlo, pure Dox, Dox–Erlo NPs, or Dox–Erlo-NPs conjugates having 1 mg Erlo and 5 μg of Dox was performed via the nose-to-brain delivery of 20 µL once per day for 14 days in four groups of male Wistar rats (*n* = 3). Different organs, viz., the liver, heart, kidney, blood, and brain were removed from each group (*n* = 3) 24 h after the last dose. The removed tissues were blotted with tissue paper, weighed, and homogenized in 1 mL of ice-cold sodium chloride solution per 1 g of tissue. Thereafter, aliquots were separated and kept at −20 °C until analysis. The Dox and Erlo contents were estimated by using HPLC, using the procedure shown in the section on HPLC methodology.

#### 4.6.12. Stability Study

This study was carried out as per ICH guidelines on three months of Dox–Erlo-NP conjugates. A stability study of the in-house-built formulation was performed to ensure the physiochemical alteration in the quality of Dox–Erlo-NP conjugates. The samples were kept in a stability chamber at an ambient temperature, 25 ± 2 °C, 65 ± 5% RH; and at a higher temperature, 40 ± 2 °C, 75 ± 5% RH, for 90 days. The evaluations were conducted at intervals of 0, 30, 60, and 90 days.

#### 4.6.13. Statistical Analysis

Quantitative data are presented as the mean ± standard deviation (SD). Statistical comparisons between different treatments were analyzed with a one-way ANOVA using Graph Pad Prism. A value of *p* < 0.05 was considered statistically significant.

## 5. Conclusions

Dox–Erlo NPs were successfully developed for the first time, prepared using a *Cinnamomum zeylanicum* biopolymer. The optimization procedure was accomplished using a three-factor and three-level Box–Behnken experimental design. The optimized composition had a biopolymer content of 2.94% *w*/*v*, a surfactant content of a 2.2% *w*/*v*, and a sonication time of 11.39 min. In this optimum composition, formulation was characterized by a particle size of 95.35 nm, a PDI of 0.102, and % drug release of 89.91%. The analytical findings confirmed that both drugs were loaded in the biopolymeric core of the NPs. The biodistribution study revealed that folate-functionalized NP conjugates showed improved Dox and Erlo transport across biological barriers and potentially enriched the drug concentration in the brain. The higher cell death in an MTT assay recorded for the NP conjugates over the drug suspension against C6 and U87 cell lines resulted in an enhanced anti-tumor efficacy. The hemolysis study demonstrated that Dox–Erlo-NP conjugates were suitable for in vivo administration. Based on the findings of the studies, it is further suggested that Dox–Erlo-NP conjugates could be an option for effective drug delivery to glioma cancer.

## 6. Patents

This work has been patented as Indian Patent Application No. 202111038933. Publication Date, 10 September 2021.

## Figures and Tables

**Table 1:** Box–Behnken design variables for formulation of Dox–Erlo NPs.

Independent Variables	Level Used		
	Low	Medium	High
	(−1)	(0)	(+1)
Polymer concentration (A), % *w*/*v*	1.00	2.00	3.00
PVA (B), % *w*/*v*	0.50	1.50	2.50
Sonication Time (C), min	3.00	7.50	12.00
**Dependent variables**			
Particle size (R1)	Minimize		
PDI (R3)	Minimize		
Drug release (R2)	Maximize		

**Table 2:** Observed responses in Box–Behnken design for Dox–Erlo NP preparations with their predicted and actual values.

Run Order	(A)	(B)	(C)	Actual Value of R1	Predicted Value of R1	Actual Value of R2	Predicted Value of R2	Actual Value of R3	Predicted Value of R3
1	3.00	1.50	12.00	121.00	128.00	89.00	89.50	0.1230	0.1208
2	2.00	0.50	3.00	230.00	230.13	78.00	77.00	0.2230	0.2253
3	2.00	1.50	7.50	180.00	190.00	71.00	75.00	0.1430	0.1332
4	3.00	0.50	7.50	240.00	236.12	71.00	72.00	0.2210	0.2235
5	1.00	1.50	3.00	159.00	152.00	65.00	64.50	0.2310	0.2332
6	1.00	1.50	12.00	100.00	96.25	63.00	63.00	0.2070	0.2117
7	2.00	2.50	3.00	227.00	230.13	57.00	58.50	0.2130	0.2132
8	2.00	1.50	7.50	201.00	190.00	74.00	75.00	0.1300	0.1332
9	2.00	1.50	7.50	189.00	190.00	79.00	75.00	0.1290	0.1332
10	3.00	1.50	3.00	170.00	173.75	83.00	83.00	0.1950	0.1903
11	2.00	1.50	7.50	200.00	190.00	76.00	75.00	0.1230	0.1332
12	2.00	2.50	12.00	130.00	129.88	73.00	74.00	0.1120	0.1097
13	1.00	0.50	7.50	136.00	142.87	59.00	60.50	0.2980	0.2935
14	1.00	2.50	7.50	156.00	159.88	45.00	44.00	0.2230	0.2205
15	3.00	2.50	7.50	127.00	120.12	79.00	77.50	0.1520	0.1565
16	2.00	0.50	12.00	232.00	228.88	68.00	66.50	0.2380	0.2377
17	2.00	1.50	7.50	180.00	190.00	75.00	75.00	0.1410	0.1332

(A) Polymer concentration, % *w*/*v*; (B) surfactant concentration, % *w*/*v*; and (C) sonication time, min. R1—Particle size; R2—drug release %; and R3—PDI.

**Table 3:** Summary results of regression analysis for response fitting of quadratic models for R1, R2, and R3.

Quadratic Model	R–Squared	Adjusted R–Squared	Predicted R–Squared	SD	% CV
Response (R1)	0.9768	0.9469	0.8327	9.94	5.68
Response (R2)	0.9740	0.9405	0.8524	2.60	3.68
Response (R3)	0.9914	0.9802	0.9502	0.0076	4.18
Regression equation of the fitted quadratic model Particle size (R1) = +190.00 + 13.37 × A − 24.75 × B − 25.38 × C − 33.25 × A × B + 2.50 × A × C − 24.75 × B × C − 46.25 × A^2^ + 21.00 × B^2^ − 6.25 × C^2^ % Drug release (R2) = +75.00 + 11.25 × A − 2.75 × B +1.25×C + 5.50 × A × B + 2.00 × A × C + 6.50 × B × C − 2.75 × A^2^ − 8.75 × B^2^ + 2.75 × C^2^ PDI (R3) = +0.1332 − 0.0335 × A − 0.0350 × B − 0.0228 × C + 0.0015 × A × B − 0.0120 × A × C − 0.0290 × B × C + 0.0414 × A^2^ + 0.489 × B^2^ + 0.0144 × C^2^					

**Table 4:** Analysis of variance (sum of square, degree of freedom, mean square, F-value, and *p*-value) for response, particle size, drug release, and PDI.

Result of the Analysis of Variance	Particle Size (nm)	Drug Release (%)	PDI
1. Regression analysis			
Sum of squares	29,090.22	1776.26	0.6131
Degree of freedom (df)	9	9	17
Mean squares	3232.25	197.36	0.0361
F-value	32.68	29.09	113.60
*p*-Value	˂0.0001	˂0.0001	˂0.0001
2. Lack-of-fit tests			
Sum of squares	270.25	13.50	0.0001
df	3	3	3
Mean squares	90.08	4.50	0.0000
F-value	0.8539	0.5294	0.5471
*p*-Value	0.5330	0.6858	0.6762
Correlation of variation (% CV)	5.68	3.68	4.18
3. Residual			
Sum of squares	692.25	47.50	0.0004
df	7	7	7
Mean squares	98.89	6.79	0.0001
SD	9.94	2.60	0.0076

**Table 5:** The optimized composition using experimental design for the development of Dox–Erlo NPs with experimental and predicted responses.

Variable Composition	Responses	Predicted Value	Experimental Value	% Error
A (2.94 % *w*/*v*)	R1	92.76 nm	95.35 ± 10.25 nm	2.79
B (2.20 % *w*/*v*)	R2	89.91%	79.203 ± 0.24%	11.90
C (11.39 min)	R3	0.102	0.109	6.8

**Table 6:** Kinetic drug release of Erlo and Dox release from Dox–Erlo-NP conjugates at pH 5.4.

**Erlo Release from Dox–Erlo-NP Conjugates at pH 5.4**		
Zero order	0.9002	1.65021
First order	0.9744	−0.0355
Higuchi matrix	0.9096	9.7543
Korsmeyer–Peppas	0.9793	3.0543
Hixson–Crowell	0.9593	0.0090
Dox release from Dox–Erlo-NP conjugates at pH 5.4		
**Model Fitting**	**R^2^**	**k**
Zero order	0.8541	1.2361
First order	0.9231	−0.0195
Higuchi Matrix	0.9233	8.3559
Korsmeyer–Peppas	0.9751	2.5251
Hixson–Crowell	0.9025	0.0056

**Table 7:** Kinetic drug release of Erlo and Dox release from Dox–Erlo-NP conjugates at pH 7.4.

**Erlo Release from Dox–Erlo-NP Conjugates at pH 7.4**		
**Model Fitting**	**R^2^**	**k**
Zero order	0.8704	1.3919
First order	0.9537	−0.0244
Higuchi Matrix	0.9190	8.8754
Korsmeyer–Peppas	0.9782	2.7330
Hixson–Crowell	0.9306	0.0067
Dox release from Dox–Erlo NPs conjugates at pH 7.4		
**Model Fitting**	**R^2^**	**k**
Zero order	0.8718	1.1629
First order	0.9294	−0.0183
Higuchi Matrix	0.9062	8.3554
Korsmeyer–Peppas	0.9709	2.9451
Hixson–Crowell	0.9125	0.0052

**Table 8:** Stability indicating data of Dox–Erlo-NP conjugates with regard to particle size, zeta potential, and % entrapment efficiency.

Sampling Period (in Days)	Particle Size (nm)		Zeta Potential (mV)		% Entrapment Efficiency	
	(25 ± 2 °C, 65 ± 5% RH)	(40 ± 2 °C, 75 ± 5% RH)	(25 ± 2 °C, 65 ± 5% RH)	(40 ± 2 °C, 75 ± 5% RH)	(25 ± 2 °C, 65 ± 5% RH)	(40 ± 2 °C, 75 ± 5% RH)
0	95.35 ± 10.23	95.35 ± 10.33	−18.1 ± 2.40	−18.1 ± 2.40	80 ± 2.3%	80 ± 4.6%
30	99.39 ± 11.03	100.46 ± 9.2	−18.3 ± 3.40	−19.3 ± 2.31	79.3 ± 3.4%	79 ± 3.2%
60	104.22 ± 13.44	106.25 ± 14.25	−19.2 ± 3.24	−20.2 ± 2.05	78 ± 3.8%	77 ± 4.4%
90	109.45 ± 12.48	115.33 ± 12.38	−21.1 ± 4.01	−20.4± 3.20	76 ± 5.3%	73 ± 3.3%

## Data Availability

Not applicable.
